# Improving Rehabilitation Research to Optimize Care and Outcomes for People with Chronic Primary Low Back Pain: Methodological and Reporting Recommendations from a WHO Systematic Review Series

**DOI:** 10.1007/s10926-023-10140-4

**Published:** 2023-11-22

**Authors:** Carol Cancelliere, Hainan Yu, Danielle Southerst, Gaelan Connell, Leslie Verville, André Bussières, Douglas P. Gross, Paulo Pereira, Silvano Mior, Andrea C. Tricco, Christine Cedraschi, Ginny Brunton, Margareta Nordin, Heather M. Shearer, Jessica J. Wong, Jill A. Hayden, Rachel Ogilvie, Dan Wang, Pierre Côté, Cesar A. Hincapié

**Affiliations:** 1 Institute for Disability and Rehabilitation Research and Faculty of Health Sciences, Ontario Tech University, Oshawa, Canada; 2 Département Chiropratique, Université du Québec à Trois-Rivières, Trois-Rivières (Québec), Canada; 3 School of Physical and Occupational Therapy, Faculty of Medicine and Health Sciences, McGill University, Québec, Canada; 4 Department of Physical Therapy, University of Alberta, Edmonton, Canada; 5 Department of Neurosurgery, Centro Hospitalar Universitário São João, Faculty of Medicine, University of Porto, Porto, Portugal; 6 Department of Research and Innovation, Canadian Memorial Chiropractic College, Toronto, Canada; 7 Li Ka Shing Knowledge Institute, St. Michael’s Hospital, Unity Health Toronto, Toronto, Canada; 8 Epidemiology Division and Institute for Health Policy, Management, and Evaluation, Dalla Lana School of Public Health, University of Toronto, Toronto, Canada; 9 Queen’s Collaboration for Health Care Quality Joanna Briggs Institute Centre of Excellence, Queen’s University, Kingston, Canada; 10 Division of General Medical Rehabilitation, Geneva University and University Hospitals, Geneva, Switzerland; 11 Division of Clinical Pharmacology and Toxicology, Multidisciplinary Pain Centre, Geneva University Hospitals, Geneva, Switzerland; 12 EPPI-Centre, UCL Institute of Education, University College London, London, England, UK; 13 Department of Health Research Methods, Evidence and Impact, Faculty of Health Sciences, McMaster University, Hamilton, Canada; 14 Departments of Orthopedic Surgery and Environmental Medicine, NYU Grossman School of Medicine, New York University, New York, USA; 15 Bloorview Research Institute, Holland Bloorview Kids Rehabilitation Hospital, Toronto, Canada; 16 Department of Community Health and Epidemiology, Dalhousie University, Halifax, Canada; 17 EBPI-UWZH Musculoskeletal Epidemiology Research Group, University of Zurich and Balgrist University Hospital, Zurich, Switzerland; 18 Epidemiology, Biostatistics and Prevention Institute (EBPI), University of Zurich, Zurich, Switzerland; 19 University Spine Centre Zurich (UWZH), Balgrist University Hospital and University of Zurich, Zurich, Switzerland

**Keywords:** Low back pain, Systematic review, Methods, Rehabilitation, Randomized controlled trials

## Abstract

Chronic primary low back pain (CPLBP) is a prevalent and disabling condition that often requires rehabilitation interventions to improve function and alleviate pain. This paper aims to advance future research, including systematic reviews and randomized controlled trials (RCTs), on CPLBP management. We provide methodological and reporting recommendations derived from our conducted systematic reviews, offering practical guidance for conducting robust research on the effectiveness of rehabilitation interventions for CPLBP. Our systematic reviews contributed to the development of a WHO clinical guideline for CPLBP. Based on our experience, we have identified methodological issues and recommendations, which are compiled in a comprehensive table and discussed systematically within established frameworks for reporting and critically appraising RCTs. In conclusion, embracing the complexity of CPLBP involves recognizing its multifactorial nature and diverse contexts and planning for varying treatment responses. By embracing this complexity and emphasizing methodological rigor, research in the field can be improved, potentially leading to better care and outcomes for individuals with CPLBP.

## Introduction

Chronic primary low back pain (CPLBP) is a complex, multifactorial, and disabling condition that affects a substantial portion of the global population [1]. In a systematic review of the global prevalence of LBP, Hoy et al. found that prevalence estimates vary widely between studies, reporting an estimated mean lifetime prevalence of 38.9% (SD 24.3) across low-, middle-, and high-income countries [2]. The overall mean prevalence ranged from 16.7% (SD 15.7) in low-income economies to 32.9% (SD 19.0) in high-income economies. CPLBP is often treated with rehabilitation interventions aimed at optimizing function and reducing pain. The World Health Organization (WHO) defines rehabilitation as a comprehensive set of interventions aimed at improving function, reducing the impact of health conditions or disabilities, and enhancing individuals’ participation in daily life [3]. These interventions should be person centered, multidisciplinary, and delivered in a coordinated manner within the broader healthcare system [3].

Researchers are dedicated to advancing the understanding of CPLBP and rehabilitation interventions to provide patients, practitioners, and policymakers with the best available evidence for guiding treatment decisions and improving outcomes. In line with this objective, our aim is to share valuable insights gained from conducting four systematic reviews on the management of CPLBP (published in this series) [4–7]. These reviews focused on examining the benefits and harms of structured and standardized education/advice, structured exercise programs, transcutaneous electrical nerve stimulation (TENS), and needling therapies for adults with CPLBP. The findings from these reviews have contributed to the development of a clinical guideline by the WHO for managing CPLBP in adults (in development at the time of this submission). To enhance future systematic reviews and research, including RCTs, on the effectiveness of rehabilitation interventions for CPLBP, we provide methodological and reporting issues, as well as recommendations.

Drawing upon our experiences conducting these systematic reviews, we have identified a range of methodological and reporting issues and recommendations that hold relevance for future CPLBP research. These issues and recommendations have been consolidated (see Table 1) and are discussed systematically, using topic areas consistent with reporting guidelines and established frameworks for critical appraisal of RCTs [8, 9]. By providing an overview of our proposed recommendations, we aim to offer practical guidance and insights that will facilitate the production of robust systematic reviews and research including RCTs on the effectiveness of rehabilitation interventions for people with complex conditions such as CPLBP.

**Table 1:** Summary of methodological and reporting issues and recommendations for systematic reviews on the effectiveness of rehabilitation interventions for CPLBP

Methodological or reporting issues identified from conducting four systematic reviews on the management of CPLBP [4–7]	Recommendations to address methodological or reporting issues
1. Knowledge user engagement in research Unclear if knowledge user perspectives were considered from study inception in the included RCTs	Include people with lived experience of CPLBP and other knowledge users (e.g., community members, clinicians, policymakers, evidence commissioners) as equal members of the research team from the inception of the research study to all research phases, to help ensure relevance and applicability of the research
2. Study population None of the included RCTs provided comprehensive information on sociodemographic, health equity indicators, clinical, and contextual factors. This resulted in the inability to conduct subgroup analyses and made it challenging to synthesize and draw conclusions about certainty of evidence using conventional methods	Describe the population (e.g., using PROGRESS-Plus or similar tool [10] [place of residence, race/ethnicity/culture/language, occupation, gender/sex, religion, education, socioeconomic status, and social capital] to identify sociodemographic characteristics using an equity lens) and its clinical characteristics (e.g., presence and type of leg pain)
3. Intervention description and selection Some interventions were insufficiently described We only included RCTs of unimodal interventions directed at the patient; however, this does not reflect clinical practice and how pain is managed in a contemporary model underpinned by the biopsychosocial approach	Describe the intervention using the template for intervention description and replication (TIDieR) checklist [11] (including comparison intervention, if applicable). This includes explaining the theoretical or conceptual underpinning of the intervention(s) (e.g., treatment mechanisms), and other factors such as whether the intervention used a trauma-informed care approach [12, 13] Consider multimodal interventions or integrated care models external to the individual (e.g., family, community/social, work/employer interventions) through a biopsychosocial lens, informed by known prognostic factors
4. Comparison intervention selection Some RCTs used no intervention as the comparison (We only included comparisons of no intervention, placebo/sham interventions, or usual care) We only included RCTs that clearly defined usual care	Select credible comparison interventions, including other interventions or credible sham if available (instead of no intervention) Provide clear definitions of usual care and other treatment comparisons
5. Blinding of participants and practitioners to the intervention Performance and detection biases were the main biases in the included RCTs	Restrict eligibility to naïve participants, and/or measure treatment credibility/expectancy and blinding and consider these in the analysis and interpretation of results
6. Outcome selection Participation outcomes were not measured in the included RCTs Unclear if participants’ perspectives and contexts were considered when selecting outcomes in the included RCTs Harms were seldomly or insufficiently reported in the included RCTs	Outcomes should include measures of meaningful participation and functioning (e.g., WHO Disability Assessment Schedule 2.0 (WHODAS 2.0) [14], Patient-Specific Functional Scale (PSFS) [15], and Global Rating of Change scales (GRC) [16] allow for flexible, individualized approaches) Individuals’ perspectives and contexts should be considered when selecting intervention outcomes. Stratify analyses based on contextual factors if possible or conduct a descriptive synthesis (vs. meta-analysis) in systematic review Comprehensively monitor and report harms in all studies of interventions (e.g., CONSORT Harms [17])
7. Analysis of intervention effects Only summary measures were reported in the included RCTs We defined minimally important differences for treatment effects using standard benchmarks and pre-defined arbitrary cut-offs informed by literature Adherence to interventions was often not reported in the included RCTs	Use responder analysis in addition to summary measures (e.g., mean differences) Enable patients to decide what a worthwhile treatment effect is for them Assess and report adherence to interventions. Assess acceptability and compliance to interventions in feasibility studies
8. Study designs Our systematic reviews included RCTs only	Consider pragmatic vs. explanatory RCTs (e.g., Pragmatic Explanatory Continuum Indicator Summary-2 (PRECIS-2) tool [18]) and other study designs (cohort, quasi-experimental, qualitative, and mixed methods studies, translational research) for a wide range of research questions concerning complex (rehabilitation) interventions (e.g., UK Medical Research Council guidance) [19, 20]. Incorporate health economics and location-specific research that focus on low- and middle-income countries and the global south
9. Grading the certainty of evidence We often downgraded the certainty of evidence in the inconsistency domain due to heterogeneous results We often downgraded the certainty of evidence in the risk of bias domain due to pooling results of trials rated as high risk of bias along with those rated as unclear or low risk of bias	Consider meta-analysis only when a high level of clinical homogeneity (e.g., populations, interventions, comparators, outcomes) is described across studies. A suitable alternative to meta-analysis is descriptive synthesis—identifying common themes, patterns, and trends across studies for a range of research questions concerning complex (rehabilitation) interventions (e.g., using Synthesis without Meta-analysis (SWiM) guideline [21]) Prioritize including low ROB studies. When low ROB studies are available, careful consideration should be given to the potential insights that high ROB studies may offer in specific contexts Consider grading studies based on *1) ROB assessment, 2) imprecision, and 3) publication bias* (i.e., certainty of evidence is not downgraded if studies are rated low ROB, have precise estimates, and there is no strong suspicion of publication bias). Less relevant: *1) inconsistency* may not be applicable for studies with heterogeneous populations (e.g., people with CPLBP and varied sociodemographic and clinical characteristics) and interventions and *2) indirectness* can be addressed by having clear and focused eligibility criteria

RCTs: randomized controlled trials, ROB: risk of bias

## Knowledge User Engagement in Research

### Methodological or Reporting Issue

It is unclear if knowledge user perspectives were considered from study inception in the included RCTs of our four systematic reviews [4–7].

Such consideration is important as people with lived experience of CPLBP and other knowledge users (e.g., community members, clinicians, policymakers, evidence commissioners) are experts of their own experiences.

### Recommendation

Knowledge users should be included in study conceptualization, research question formulation, study design, outcome selection and measurement (including identifying and prioritizing contextual factors that may influence outcomes [22]), applicability of the intervention, shaping data collection methods, recruitment strategies, data collection, and interpretation and dissemination of findings [23]. Engaging knowledge users in research offers numerous advantages, such as aligning research with their values, preferences, and needs, resulting in outcomes that are more patient centered and relevant [24]. Furthermore, the invaluable insights and lived experiences of knowledge users can significantly enhance the relevance, quality, and applicability of research design, enabling it to effectively address real-world challenges and yield meaningful results [19]. This engagement can also contribute to the development of implementation strategies for clinical practice and policy. Creating a safe and inclusive space for knowledge users to participate fully is paramount. It is important to ensure their meaningful engagement, fair compensation for their contributions, and the evaluation of knowledge user engagement processes to mitigate any potential harm [25]. Additionally, it is crucial to strive for representation across diverse settings and economies to ensure that the perspectives and needs of all stakeholders are considered.

## Study Population

### Methodological or Reporting Issue

None of the RCTs included in our systematic reviews provided comprehensive information on sociodemographic, health equity indicators, clinical, and contextual factors. Therefore, we were unable to determine whether the intervention groups tested in the included RCTs were comparable on important factors. We were unable to conduct pre-specified subgroup analyses and generate specific insights for informing clinical practice with respect to different patient profiles, for example, with respect to the presence and type of leg pain, race/ethnicity, age, and gender (sex was measured, but not gender, although these variables are often conflated).

### Recommendation

Regardless of study design, the study population should be clearly described because sociodemographic, clinical, and contextual characteristics are associated with outcomes, and this may help to understand health inequities, which is a strategic priority of most health organizations [26]. This information is necessary to determine whether the intervention groups are comparable or different and may provide readers with a better understanding for whom and to what contexts research findings may apply.

At present, there is a lack of consensus regarding the inclusion of equity measures in pain trials. Although ongoing efforts are underway to address this issue, there appears to be a disconnect between clinical outcomes, such as those outlined in the IMMPACT (Initiative on Methods, Measurement, and Pain Assessment in Clinical Trials) [27] and other guidelines [28] and the actual needs and perspectives of individuals from diverse backgrounds, including those in low- and middle-income countries. This discrepancy reflects a predominant focus on high-income country perspectives, which may not fully capture the lived experiences and equity considerations necessary for a comprehensive understanding of pain interventions.

The PROGRESS-Plus tool can help researchers describe the population in individual studies and guide data extraction in systematic reviews from an equity lens [10]. *PROGRESS* refers to place of residence, race/ethnicity/culture/language, occupation, gender/sex, religion, education, socioeconomic status, and social capital. Plus refers to 1) personal characteristics associated with discrimination (e.g., age, disability), 2) features of relationships (e.g., smoking parents, excluded from school), and 3) time-dependent relationships (instances where a person may be temporarily at a disadvantage related to their health and well-being, such as challenges that individuals may face during the transition from hospital care back to their everyday lives).

## Intervention Description and Selection

### Methodological or Reporting Issue

In our reviews, the interventions were not sufficiently described in 14 (17%) of the 82 included RCTs to allow for adequate interpretation and comparison of findings among studies or to inform clinical decision-making. Details missing included how the intervention was carried out and by whom (e.g., practitioner type).

### Recommendation

Accurate and comprehensive reporting of a rehabilitation intervention, for example, using the template for intervention description and replication (TIDieR) checklist [11] (e.g., type of intervention, program duration, number and duration of treatment sessions, profession of practitioner who delivered the intervention, and mode of delivery) is crucial for properly understanding the findings of a study [29] for study replication and for implementing an intervention in clinical practice. In so doing, when study results yield null findings, it can indicate an ineffective intervention, but it can also be attributed to other factors, such as inadequate delivery of the intervention. As with the active intervention, it is also important to adequately describe any comparison interventions using the TIDieR template [11, 30, 31].

The intent of an intervention should be clearly explained and its underlying mechanism of action rationalized based on theory. A treatment theory framework, which explains how interventions work and the mechanisms by which they produce their effects within a given context, can be used for this purpose [29] [32]. Using a treatment theory framework, interventions are grounded in scientific understanding and empirical evidence, providing a clear rationale for their implementation, thus helping to ensure that interventions are not selected arbitrarily [29]. Such frameworks should outline the potential causal pathways and mechanisms through which the intervention is expected to have a therapeutic effect. Treatment theory also incorporates patient characteristics, values, and goals, as well as the cost implications of different interventions. It recognizes that interventions may involve active administration by healthcare practitioners or involve other social partners, while some may be self-administered by patients at home. Through shared decision-making, the evidence, patient preferences, local circumstances, and the costs and resources associated with specific interventions can be considered when making informed decisions about an intervention’s feasibility and practicality.

For complex interventions like rehabilitation for CPLBP, which may involve multiple components and target different mechanisms tailored to individual needs, designing, selecting, and reporting studies based on a treatment theory framework becomes particularly relevant. Such interventions should also target known prognostic factors of chronicity or other poor outcomes, further emphasizing the importance of treatment theory in guiding evidence-based care [29]. By integrating treatment theory with the understanding of prognostic factors, researchers and practitioners can ensure that interventions are well founded and aligned with the goals of improving patient outcomes.

### Methodological or Reporting Issue

We only included RCTs of unimodal interventions directed at the patient in our systematic reviews [4–7]; however, this does not reflect clinical practice and how pain is managed in a contemporary model underpinned by the biopsychosocial approach. We excluded RCTs of multimodal interventions if the specific attributable effect of the single intervention could not be isolated (e.g., exercise + treatment B vs. treatment B alone). We found that the intervention effects across the reviews were small to modest at best. However, this should not be surprising as CPLBP is a complex condition and disability can persist due to a variety of factors [19]. These can include physical factors, such as a sedentary lifestyle, occupational loading, previous injuries, and comorbid conditions; psychological factors, such as fear, depression, low job satisfaction, or recovery expectations [33]; social factors, such as poverty, poor social relationships at home or work, or lack of access to healthcare, barriers to participation in the physical, social and occupational environment; patient factors, such as inappropriate treatment expectations and unhelpful beliefs; or care history factors, such as instances where individuals may have received inadequate care and advice through their healthcare journey [34]. Thus, implementing and assessing unimodal interventions are likely to yield only modest effects at best, as they inadequately address the multifactorial etiology that is common in CPLBP.

### Recommendation

Integrated multimodal interventions that are selected and sequenced to match the needs and preferences of the person, informed by a biopsychosocial perspective and framed within the WHO International Classification of Functioning, Disability and Health (ICF) [35], are likely to be effective for complex conditions, like CPLBP, despite being challenging to implement and evaluate. These types of trials are currently limited in the pain literature [36]; however, the recent RESTORE trial (cognitive functional therapy with or without movement sensor biofeedback versus usual care for chronic, disabling low back pain) provides an example of this type of intervention [37].

Interventions encompassing factors external to the individual person could also be considered. Such interventions may involve family, community, work, trauma-informed care approaches, or other social components, thereby recognizing the influence of broader contextual factors on the condition and its management [26, 37]. The trauma-informed care approach is a healthcare strategy that acknowledges the widespread impact of trauma and seeks to understand and respond to the effects of trauma in patients’ lives [13]. It involves viewing patients through the lens of their life experiences, particularly traumatic ones, rather than focusing solely on their current symptoms or behaviors. The goal is to provide care in a way that avoids re-traumatization and promotes healing and resilience.

This understanding suggests that pragmatic trials, which focus on real-world effectiveness and implementation, are necessary to complement the results of explanatory trials to evaluate the effectiveness of rehabilitation interventions. Pragmatic trials can capture the complex nature of interventions and their impact in real-life settings, accounting for the diverse factors that influence outcomes in complex conditions [38]. However, this was outside the scope of our reviews and should be considered for future research.

## Comparison Intervention Selection

### Methodological or Reporting Issue

Our systematic reviews included comparisons of no interventions, placebo/sham interventions, and usual care. Across three of our four reviews (on standardized and structured education/advice, TENS, needling therapies) [4, 6, 7], 13 of 69 included RCTs (19%) compared the active intervention with no intervention (i.e., no therapeutic component, for example, waitlist control). In our review on structured exercise programs [5], RCTs rated as overall high risk of bias were not included in our primary analysis and notably, all the RCTs that used a ‘no intervention’ comparison were rated as high risk of bias. Participants who do not receive any treatment may report worse outcomes compared to those who receive the active intervention simply because they did not *expect* improvement without treatment [39], resulting in the illusion that the experimental intervention is effective.

### Recommendation

Non-specific intervention effects, such as expectations of improvement after an intervention, are associated with outcomes [31, 33]. Therefore, it is important for research teams to include credible comparison interventions, such as other interventions or a credible sham, if available, instead of no treatment (e.g., waitlist controls).

Notably, many rehabilitation interventions are studied in comparative effectiveness studies rather than sham-controlled studies. There are a few reasons for this. Sham-controlled studies typically involve a placebo or inactive treatment that mimics the active intervention. However, in rehabilitation interventions, the active treatment may involve a combination of physical exercises, therapies, and patient education, making it challenging to create an appropriate sham control. Second, rehabilitation interventions are often tailored to the individual needs and goals of the patient. The focus is on optimizing function and improving quality of life rather than simply treating a specific condition or symptom. This personalized approach makes it difficult to have a standardized sham control that can adequately mimic the individualized nature of the intervention. Third, ethical considerations come into play. In some cases, withholding or providing a sham treatment to individuals with genuine rehabilitation needs may not be ethically justifiable. It may be more appropriate to compare different active treatments or variations of standard care to evaluate their comparative effectiveness. Lastly, comparative effectiveness studies may provide valuable insights into real-world clinical practice and allow for the evaluation of interventions in diverse populations and settings. They may provide information on the relative benefits, risks, and costs of different rehabilitation interventions, helping clinicians and patients make informed decisions.

### Methodological or Reporting Issue

We encountered the issue of inconsistent or unclear definitions of “usual care” across RCTs. To ensure a consistent comparison, we specifically included RCTs where the definition of usual care clearly stated that it referred to the standard treatment or interventions received by patients under usual clinical practice conditions, typically provided by a general medical practitioner.

### Recommendation

Usual care serves as a reference point for assessing the added value or impact of an experimental intervention. We recognize that usual care may be defined differently depending on the region or context. The availability of clear definitions of usual care enables comparability of study findings across different settings and populations which is essential for systematic reviews, enables practitioners to make informed decisions about intervention adoption, and it assists policymakers in developing guidelines and policies that align with real-world practice and address the needs of the population.

## Blinding of Participants and Practitioners to the Intervention

### Methodological or Reporting Issue

Performance bias (occurs with unequal care between groups) and detection bias (due to outcome assessors’ knowledge of the allocated intervention) were the main biases in the included RCTs in our reviews, with 77 (94%) and 60 (73%) of 82 trials having unclear or high risk of bias in these domains, respectively (note that for the structured exercise programs review, we only referred to the 13 low or unclear risk of bias RCTs in the primary analysis). In RCTs of rehabilitation interventions, such as exercise, workplace, or educational interventions, it may be impossible to blind participants and providers to the intervention. This can lead to over- or under-estimated intervention effects from performance bias and detection bias. Note that participants are the outcome assessors when self-reported outcome measurements are used, which was the case in all included RCTs in our reviews [9, 40–42].

### Recommendation

To deal with these issues, eligibility in RCTs can be restricted to participants naïve to the studied interventions. Alternatively, in RCTs or feasibility studies conducted prior to RCTs, treatment credibility and expectancy can be measured (in participants and practitioners) using the credibility/expectancy questionnaire [43] and blinding can be measured using the Bang Blinding Index, for example, which measures the adequacy of blinding [44]. For instance, in a feasibility study assessing a back strengthening program vs. a back strengthening program + medication, participants and providers could be provided with information about these two interventions. They could then be asked to rate their expectations and beliefs about the effectiveness of each intervention, and participants could guess which treatment group they were assigned to using the tools [43, 44]. Gathering this information on treatment credibility/expectancy and the success of blinding can help researchers gauge the potential for bias and develop strategies to mitigate it in subsequent RCTs. This information can also be considered in the analysis (e.g., controlling for credibility/expectancy) and explored and interpreted to provide more context on these potential biases and the implications on intervention effect estimates (e.g., whether the bias likely leads to under- or over-estimating intervention effects).

## Outcome Selection

### Methodological or Reporting Issue

Outcome measures used in the included RCTs in our reviews typically measure impairments (e.g., physical such as pain and cognitive) or specific functional limitations (e.g., Oswestry Disability Index, Roland-Morris Disability Questionnaire). These outcome measures align with LBP core outcome set recommendations [28]. However, available pain measuring tools may be inappropriate in some populations considering distinct cultural beliefs regarding the meaning, origin, and role of pain, which can affect how a person interprets and perceives pain [45]. Additionally, outcomes related to equity or the individual’s ability to participate in activities that are meaningful to them despite impairments (e.g., paid and unpaid work, recreational or leisure activities, community activities or events), as conceptualized by the WHO International Classification of Functioning, Disability and Health (ICF) model [35, 46], were not measured. Guideline developers, including the WHO Guideline Development Group, often seek data on important outcomes for informing their recommendations. However, there is a disconnect between the outcomes desired by guideline panels and the outcomes reported in RCTs. RCTs frequently fail to provide the specific outcomes that guideline developers require, creating a gap between the information they need and what the trials actually deliver. This disconnect poses a challenge in aligning research evidence with the development of comprehensive and relevant guidelines.

### Recommendation

For people with CPLBP, the outcomes should primarily focus on meaningful participation and functioning. Thus, in addition to condition-specific questionnaires such as those recommended by the LBP core outcome set [28], we recommend other outcome measures be considered that reflect patients’ preferences, values, goals, culture, and experiences. People should be empowered to identify the outcomes that are personally meaningful to them, ensuring that the assessment captures their individual perspectives and priorities (this can include but is not limited to patient-reported outcomes and experience measures [PROMs and PREMs]) [47]. Of note, patient experience measures were not commissioned to be included in our reviews. Such outcomes may include the 12-item WHODAS 2.0 (WHO Disability Assessment Schedule 2.0) [14], which is linked to the International Classification of Functioning, Disability, and Health (ICF), providing a universally applicable approach that considers individual perspectives, diverse impacts of health conditions and allows for customization based on priorities and experiences. The Patient-Specific Function Scale (PSFS) [15] offers an individualized and patient-centered approach, allowing individuals to prioritize and assess activities or participation that are most important to them. The Global Rating of Change (GRC) scales [16] focus on overall change and enable individuals to evaluate the meaningful changes in their functional status, providing valuable insights into their progress beyond specific activities or domains. These patient-centered approaches enhance the assessment of disability and functioning and provide meaningful outcomes in rehabilitation that can be applied across different cultural contexts.

People with CPLBP may value outcomes such as increased self-efficacy, increased knowledge about their condition, and improved ability to cope and manage their symptoms. Indeed, a recent conceptualization of health is ‘the ability to adapt and to self-manage, in the face of social, physical and emotional challenges’ [48]. For example, clinicians may consider the option of TENS for people with CPLBP as a method to improve self-efficacy (patients can use this as one method to take some control over their pain while at home) or as a relaxation method before other interventions of a multimodal program are provided. Rarely do clinicians use TENS as the sole intervention for patients with CPLBP. Thus, we suggest that there is value in measuring these additional outcomes in future research.

### Methodological or Reporting Issue

Our reviews encompassed RCTs conducted in multiple countries. Yet, it is unclear if participants’ perspectives and contexts were considered when selecting or interpreting outcomes in the included RCTs. Further, the lack of reporting participants’ sociodemographic, health equity indicators, and contextual characteristics resulted in the inability to conduct subgroup analyses and made it challenging to synthesize and draw conclusions about certainty of evidence using conventional methods.

Contextual factors like cultural variations and individual preferences can influence treatment outcomes [49]. The impact and meaning of living with or without a disability can differ among individuals based on their cultural backgrounds and personal circumstances [50, 51]. These cultural nuances manifest in attitudes toward disability, support system availability, and societal expectations of independence or interdependence. Consequently, individuals with disabilities navigate their experiences, cope with challenges, and seek support in culturally influenced ways [52].

Contextual factors also interact with treatments, modifying their effects or mediating patient responses [53]. Treatment effectiveness can vary across cultural contexts, influenced by factors, such as belief systems, spirituality, religion, attitudes, and social support systems [50, 51]. As a result, the conventional approach of combining study results in meta-analyses may oversimplify the complexities of interventions in diverse contexts. Understanding these contextual factors also aids practitioners in adjusting their expectations regarding the benefits of clinical rehabilitation interventions and designing complex interventions that address contextual considerations.

### Recommendation

We recommend conducting stratified analyses, if feasible, or employing a descriptive synthesis approach in systematic reviews. This involves describing the study participants, interventions, and outcomes in detail and providing comprehensive information to readers about the applicability of the results to specific contexts. By stratifying analyses based on contextual factors or employing descriptive synthesis, a more nuanced understanding can be gained, allowing for better interpretation of the study findings and their potential relevance to different populations or settings.

### Methodological or Reporting Issue

Only 12 (15%) of the 82 included RCTs across our reviews reported on adverse events. While the focus is often placed on evaluating the effectiveness (or benefits) of interventions, it is equally important to measure and report their potential harms or adverse effects [17].

### Recommendation

Comprehensively monitoring and reporting harms of interventions is essential for patient safety, informed decision-making, balancing risks and benefits, identifying rare or delayed harms, improving intervention safety, and informing regulatory and policy decisions [17].

## Analysis of Intervention Effects

### Methodological or Reporting Issue

In the RCTs included in our reviews, responder analysis, which measures the proportion of participants achieving a pre-defined level of improvement, was not reported [54]; only summary measures were reported. Responder analysis offers advantages by considering individual variability in treatment response and allowing for tailored treatment approaches. However, it also has disadvantages, such as varying definitions of responders and potential loss of information [55]. Moreover, responder analysis requires that eligibility be restricted to those who can respond, i.e., individuals with a high enough level of the outcome at baseline to allow a realistic change.

### Recommendation

Recognizing that there are advantages and disadvantages to using summary measures and responder analysis, we suggest that future studies report both. By combining the overall intervention effects with individual-level responses, researchers can gain valuable insights into the magnitude and variability of treatment outcomes.

### Methodological or Reporting Issue

In our reviews, we defined minimally important differences for treatment effects using standard benchmarks (e.g., minimal clinically important difference (MCID) of outcome measure) and pre-defined arbitrary cut-offs (e.g., 10% improvement in outcome measure if MCID is unknown) informed by literature. Authors often dismiss treatments if their effects do not surpass a standard benchmark, such as a specific threshold on a pain scale. However, this approach oversimplifies the complexity of the clinical encounter and the preferences of the patient [56].

### Recommendation

In the context of clinical trials and systematic reviews, researchers should reconsider the conventional understanding of the “smallest worthwhile effect” [56]. Rather than viewing the smallest worthwhile effect as a fixed attribute of the measure, it should be recognized as a context-specific concept, influenced by a multitude of factors such as the patient’s baseline health status, the cost and risk associated with the treatment, and its impact on other aspects of health-related quality of life. The prevalent benchmark approach, which often leads to inconsistent decisions across studies and marginalizes the patient’s role in decision-making, warrants re-evaluation. Instead, researchers and practitioners are encouraged to adopt a more nuanced approach. This involves communicating the magnitude, precision, and certainty of the treatment effect estimate, rather than resorting to a binary categorization of results as ‘use treatment’ or ‘don't use treatment.’ This approach fosters a more comprehensive understanding of the treatment’s impact and facilitates shared decision-making between practitioners and patients [56].

### Methodological or Reporting Issue

Across our reviews, adherence to the interventions was not reported by 54 (66%) of the 82 included RCTs. Understanding adherence to interventions is important for assessing treatment effectiveness [57]. In the case of exercise interventions, adherence encompasses factors such as the frequency, duration, intensity, and progression of exercise sessions, as well as compliance with any additional instructions or guidelines provided. This is important because high adherence to exercise interventions may be associated with improved functional outcomes, symptom management, and overall health benefits. Several factors can influence adherence to exercise interventions. These include individual characteristics (e.g., motivation, self-efficacy, health beliefs), environmental factors (e.g., access to facilities, social support), and intervention-related factors (e.g., program design, flexibility, variety) [58].

### Recommendation

Researchers should assess intervention adherence and factors related to adherence. Regarding exercise, assessing adherence can be done through various methods, including self-report measures, activity trackers, exercise logs, and direct observation. Understanding these factors is essential for supporting long-term adherence to exercise interventions. To enhance adherence, interventions should be designed with patient-centered principles in mind, considering individual preferences, capabilities, and lifestyle factors. Providing clear instructions, setting realistic goals, fostering a supportive environment, regular monitoring, feedback, and personalized interventions tailored to address barriers can further enhance adherence [59]. Furthermore, it is important to assess the acceptability and compliance to interventions in feasibility studies. Note that feasibility studies were considered outside the scope of our reviews and thus excluded.

## Study Designs

### Methodological or Reporting Issue

Our systematic reviews included RCTs only, as commissioned by the WHO. While RCTs are beneficial, especially in estimating the average causal effect of an intervention in a group of individuals, there are challenges associated with using this design when studying rehabilitation interventions. These include 1) rehabilitation interventions, such as structured exercise and educational programs, are ideally tailored to the individual, making it difficult to standardize the intervention across a group of participants (which can be addressed with pragmatic RCTs); 2) key methodological strengths of RCTs, such as blinding, may be very challenging to implement when studying rehabilitation interventions; 3) RCTs cannot address ‘how’ or ‘why’ interventions are, or are not, effective; and 4) RCTs may provide results that are irrelevant to the specific healthcare decisions individuals, health professionals, and other decision-makers need to make [60, 61]. This is because RCTs typically aim to evaluate treatment effects in a controlled research setting, which may differ from real-world clinical practice and patient preferences.

### Recommendation

The Pragmatic Explanatory Continuum Indicator Summary-2 (PRECIS-2) tool may help reduce research waste by enabling trialists to match research design decisions, and the usefulness of subsequent results, to the intended knowledge users [18]. The PRECIS-2 tool facilitates the design of pragmatic RCTs (test the effectiveness of an intervention in real-life routine clinical practice conditions) versus explanatory RCTs (test whether an intervention works under optimal conditions) [62].

To ensure a more comprehensive understanding of complex and social interventions for conditions, like CPLBP, we recommend conducting research involving additional study designs such as cohort (including quasi-experimental design), qualitative, mixed methods, and implementation studies (e.g., UK Medical Research Council guidance) depending on the research question [19, 20]. These alternative designs can complement the evidence obtained from RCTs, providing a more holistic perspective on benefits and harms. For instance, qualitative and mixed methods studies can help to better understand the context of patients’ experiences and bring deeper meaning to outcomes. It is also important to consider focusing scarce research resources on translational research, which aims to bridge the gap between research findings and their implementation in clinical practice [63]. Notably, the current allocation of research resources is heavily skewed toward conducting RCTs, systematic reviews, and clinical practice guidelines, with limited funding available for implementation studies. Despite the substantial number of RCTs (> 3500), systematic reviews (> 550), and clinical practice guidelines (> 150) conducted, the resulting advancements in knowledge and clinical benefit have been marginal, particularly for CPLBP [64, 65]. Therefore, there is a need to reallocate research resources to prioritize implementation studies, which focus on understanding how to effectively integrate research findings into real-world practice settings.

## Grading the Certainty of Evidence

### Methodological or Reporting Issue

In each of our reviews, we consistently downgraded the certainty of evidence in the inconsistency domain due to heterogeneous results across comparisons.

We used the GRADE (Grading of Recommendations, Assessment, Development and Evaluation) approach in our systematic reviews [66]. GRADE focuses on intervention effectiveness, is used to classify the certainty of evidence in systematic reviews and clinical practice guidelines, and is endorsed by organizations around the world [67]. The method involves grading effect estimates (most often pooled from meta-analyses) in five domains (see Table 2) [68]. In our reviews, we primarily employed meta-analysis with the objective of amalgamating studies that exhibited clinical, statistical, and methodological homogeneity. However, we encountered instances of statistical and methodological heterogeneity in several of our meta-analyses.

**Table 2:** Grading the certainty of evidence using GRADE (Grading of Recommendations, Assessment, Development and Evaluation) [66]

GRADE domain	Explanation
Risk of bias	Evaluates the extent to which studies included in the review have been conducted with high methodological quality and are free from biases that could influence the results In randomized controlled trials, factors assessed include randomization and concealment process, blinding of participants and intervention providers, blinding of outcome assessment, missing outcome data, outcome measurement, and selective outcome reporting
Inconsistency	Examines the degree of heterogeneity in the results across studies. It considers whether there is consistency or inconsistency in the direction and magnitude of treatment effects across different studies Inconsistency can arise due to differences in populations, interventions, outcome measures, or study design Inconsistency in results is not always a negative aspect. It can provide valuable insights into the phenomenon being studied. Inconsistency can suggest the presence of effect modification (i.e., the effect of an intervention may be influenced by certain factors). These factors (i.e., effect modifiers) can include participant characteristics, contextual factors, or specific conditions. When effect modification occurs, the relationship between the intervention and outcome may vary depending on the presence or levels of these effect modifiers
Indirectness	Evaluates the extent to which the available evidence directly addresses the specific clinical question of interest. It considers whether there are limitations in the directness or applicability of the evidence to the target population, intervention, or outcomes of interest
Imprecision	Evaluates the precision of the estimated treatment effects. It considers the confidence intervals around the effect estimates and the sample sizes of the included studies. If the confidence intervals are wide or the sample sizes are small, it suggests imprecision and a higher degree of uncertainty in the estimates
Publication bias	Assesses the potential for bias resulting from selective publication of studies based on their findings. It considers whether the available evidence is likely to be affected by the omission of studies with negative or null results, which could lead to an overestimation of treatment effects

GRADE is primarily designed for assessing and synthesizing pooled results from evidence from multiple studies. However, in our experience, meta-analysis and grading the subsequent pooled results may not always be amenable in the rehabilitation field because the populations and interventions are often highly varied, leading to heterogeneous results. While the interventions may be reported similarly, the individualized components may differ; and important individual, clinical, and contextual factors of the participant samples are often not described. These issues can contribute to high heterogeneity of results in reviews, which leads to downgrading the certainty of evidence in the inconsistency domain. It is appropriate to use GRADE in the assessment of drug trials, for example, whereby the intervention is standardized, and outcomes may not be as influenced by contextual factors as they may be with rehabilitation interventions.

### Recommendation

A descriptive synthesis approach, describing study results without pooling the effect estimates (e.g., using Synthesis without Meta-analysis (SWiM) guidelines [21]), is a suitable alternative to meta-analysis when dealing with clinically heterogeneous studies within a systematic review [21, 69]. Rather than attempting to pool the data and calculate a single effect size, it aims to identify common themes, patterns, and trends across the studies. The synthesis involves a systematic and rigorous assessment of the quality, relevance, and applicability of individual studies [21]. The studies are typically graded or categorized based on their methodological quality (e.g., low vs. high risk of bias) and the strength and precision of their findings. The synthesis involves summarizing the results of the studies, identifying consistencies or discrepancies in the findings, exploring potential reasons for variation, and drawing conclusions to help inform clinical decision-making based on the overall body of evidence.

### Methodological or Reporting Issue

We consistently downgraded the certainty of evidence in the risk of bias domain due to pooling results of RCTs rated as high risk of bias (ROB) along with those rated as unclear or low ROB across comparisons.

Pooling the results of high and low ROB RCTs is problematic because this leads to downgrading the certainty of evidence and missing or diluting the high-quality evidence. For example, in our review of structured exercise programs [5], our primary analysis that excluded high ROB RCTs demonstrated moderate certainty evidence that exercise reduces pain and functional limitations. However, in our supplementary analysis that included all RCTs regardless of ROB, the certainty of evidence dropped to low or very low. Thus, focusing on low ROB studies when available may provide more moderate to high-certainty evidence, rather than the low to very low certainty evidence we often see in systematic reviews of rehabilitation interventions.

### Recommendation

We suggest prioritizing the inclusion of low ROB studies, since the certainty of the point estimate is related to the ROB of the individual studies. Indeed, the universal criterion to determine the confidence of research findings has been the high internal validity of studies along with the reproducibility of results across studies with reasonably similar populations, interventions, comparison interventions, and outcomes [70]. However, when low ROB studies are available, careful consideration should be given to the potential insights that high ROB studies may offer. While high ROB studies have limitations, they may provide some insights for specific contexts, especially in cases where alternative evidence is scarce or unavailable; this includes careful consideration of how the high ROB is likely to affect results (e.g., likely to overestimate or underestimate treatment effects).

For grading the certainty of evidence regarding the effectiveness of rehabilitation interventions we suggest to (1) consider meta-analysis only when a high level of clinical homogeneity (e.g., populations, interventions, comparators, outcomes) is described across studies. A suitable alternative to meta-analysis is descriptive synthesis; (2) consider grading studies based on *ROB assessment, imprecision, and publication bias* (i.e., certainty of evidence is not downgraded if studies are rated low ROB, have precise estimates, and there is no strong suspicion of publication bias); and (3) focus on the best available evidence (i.e., low ROB studies) and exclude high ROB studies when low ROB studies are available. With respect to the remaining two GRADE domains, *inconsistency* may not be applicable for studies with heterogeneous populations (e.g., people with CPLBP and varied sociodemographic and clinical characteristics) and interventions and *indirectness* can be addressed by having clear and focused eligibility criteria in the systematic review.

## Conclusion

This research article emphasizes the importance of utilizing evidence-based systematic reviews to advance our understanding of clinical research regarding CPLBP. Despite the considerable attention given to CPLBP, the disability burden remains high and continues to increase globally. Our identification of methodological and reporting issues, along with the research recommendations we provide for rehabilitation in CPLBP, address the need for comprehensive approaches when studying rehabilitation interventions.

CPLBP is a complex condition, but often, studies and reviews oversimplify by seeking a single, uncomplicated answer that does not represent the complexity of the problem. Embracing complexity requires acknowledging the multifactorial nature of the condition and the diverse contexts in which it manifests. It necessitates an understanding that complex phenomena cannot be reduced to simplistic explanations or approached with one-size-fits-all interventions.

By accepting that complex phenomena yield varying treatment responses and acknowledging and planning for such complexity with high methodological rigor, we can improve research in this field aimed to improve healthcare and important outcomes for individuals living with CPLBP.
